# Timing of Ibuprofen Use and Bone Mineral Density Adaptations to Exercise Training

**DOI:** 10.1002/jbmr.24

**Published:** 2010-01-14

**Authors:** Wendy M Kohrt, Daniel W Barry, Rachael E Van Pelt, Catherine M Jankowski, Pamela Wolfe, Robert S Schwartz

**Affiliations:** 1 Department of Medicine, Division of Geriatric Medicine, University of Colorado DenverDenver, CO, USA; 2 Department of Medicine, Division of General Internal Medicine, University of Colorado DenverDenver, CO, USA; 3 Department of Preventive Medicine and Biometrics, University of Colorado DenverDenver, CO, USA

**Keywords:** exercise training, bone mineral density, nonsteroidal anti-inflammatory drugs, cyclooxygenase, prostaglandins

## Abstract

Prostaglandins (PGs) are essential signaling factors in bone mechanotransduction. In animals, inhibition of the enzyme responsible for PG synthesis (cyclooxygenase) by nonsteroidal anti-inflammatory drugs (NSAIDs) blocks the bone-formation response to loading when administered before, but not immediately after, loading. The aim of this proof-of-concept study was to determine whether the timing of NSAID use influences bone mineral density (BMD) adaptations to exercise in humans. Healthy premenopausal women (*n* = 73) aged 21 to 40 years completed a supervised 9-month weight-bearing exercise training program. They were randomized to take (1) ibuprofen (400 mg) before exercise, placebo after (IBUP/PLAC), (2) placebo before, ibuprofen after (PLAC/IBUP), or (3) placebo before and after (PLAC/PLAC) exercise. Relative changes in hip and lumbar spine BMD from before to after exercise training were assessed using a Hologic Delphi-W dual-energy X-ray absorptiometry (DXA) instrument. Because this was the first study to evaluate whether ibuprofen use affects skeletal adaptations to exercise, only women who were compliant with exercise were included in the primary analyses (IBUP/PLAC, *n* = 17; PLAC/PLAC, *n* = 23; and PLAC/IBUP, *n* = 14). There was a significant effect of drug treatment, adjusted for baseline BMD, on the BMD response to exercise for regions of the hip (total, *p* < .001; neck, *p* = .026; trochanter, *p* = .040; shaft, *p* = .019) but not the spine (*p* = .242). The largest increases in BMD occurred in the group that took ibuprofen after exercise. Total-hip BMD changes averaged –0.2% ± 1.3%, 0.4% ± 1.8%, and 2.1% ± 1.7% in the IBUP/PLAC, PLAC/PLAC, and PLAC/IBUP groups, respectively. This preliminary study suggests that taking NSAIDs after exercise enhances the adaptive response of BMD to exercise, whereas taking NSAIDs before may impair the adaptive response. © 2010 American Society for Bone and Mineral Research.

## Introduction

Prostaglandin E_2_ (PGE_2_) increases in bone in response to mechanical loading and appears to be an essential intermediate in the signaling pathway for bone formation (i.e., mechanotransduction).(6,34,36) The key enzyme involved in the production of PGE_2_ and other prostaglandins is cyclooxygenase (COX). Inhibition of COX with nonsteroidal anti-inflammatory drugs (NSAIDs) markedly diminishes the bone-formation response to mechanical stress in laboratory animals and cultured osteoblasts.(5,7–9,20) This effect has been observed in response to both nonselective(5,7–9) and COX-2 selective inhibitors,(9,20) which is consistent with the observation that mechanotransduction is mediated primarily through the activation of COX-2.(2) Importantly, the timing of NSAID administration appears to be a key determinant of the bone-formation response. Formation is significantly attenuated when NSAIDs are administered before mechanical loading, but not when they are administered after.(7,20)

An important limitation of these studies is that they evaluated the bone-formation response to acute mechanical loading only. Thus it is not clear whether the usual effects of repeated bouts of mechanical loading (i.e., exercise training) to increase bone mineral density (BMD) and strength are impaired by NSAID use. Chronic exposure to NSAIDs has been found to have deleterious effects on bone in rats restricted to usual cage activity,(30,32) although this is not a consistent observation.(12) Because PGE_2_ plays a role in the activation of both bone formation and resorption, chronic NSAID use could have antiformation and/or antiresorptive actions, and the net effect on BMD could be beneficial, detrimental, or neutral.(28) In this context, it is not surprising that the human cohort studies that have examined the associations of NSAID use with BMD, markers of bone turnover, or fracture risk have yielded mixed results.(3,4,19,23,29,35) Importantly, a consistent finding among studies of animals is that NSAIDs markedly diminish the bone-formation response to mechanical stress when administered before loading.

Because there have been no studies, in animals or humans, of the effects of NSAID use on skeletal adaptations to exercise, this study was considered a preliminary, proof-of-concept investigation. The primary aim was to determine whether ibuprofen, a commonly used nonselective COX inhibitor, attenuates the BMD adaptations to exercise training. A second aim was to determine whether the timing of ibuprofen use (i.e., before versus after exercise) influences the adaptive response. Based on the studies of the timing of NSAID administration on the bone-formation response to a single loading bout in animals,(7,20) we hypothesized that taking ibuprofen before exercise sessions would attenuate the increases in BMD in response to exercise training when compared with taking ibuprofen after exercise sessions or with placebo treatment.

## Materials and Methods

### Study design and participants

This was a randomized, double-blinded, placebo-controlled study of the effects of ibuprofen use and the timing of use relative to performance of exercise on the BMD response to a 9-month exercise training program. Although conducted as a randomized, controlled trial, this was a proof-of-concept study to determine whether there is translation of the skeletal effects of NSAIDS in rodents(7,20) to human physiology. Volunteers provided written informed consent to participate, and the study was approved by the Colorado Multiple Institutional Review Board.

The participants were healthy, eumenorrheic women aged 21 to 40 years. Eumenorrheic status was defined as having a menstrual cycle length of 25 to 31 days and at least 10 cycles in the preceding year. Women on progestin-only contraceptive therapy were excluded, but other hormonal contraceptive therapy was allowed. Other inclusion criteria were exercising less than 3 days per week at moderate to high intensity; body mass index (BMI) of less than 30 kg/m^2^; nonsmoker for more than 2 years; not pregnant or lactating; no NSAID intolerance or sensitivity; typical NSAID use less than 3 days per month; no use of drugs that affect bone metabolism; serum thyroid-stimulating hormone concentration of 0.5 to 5.0 µU/mL; hematocrit of 30% or greater; serum creatinine concentration of less than 1.4 mg/dL; no history of ulcers, gastrointestinal bleeding, gastroesophageal reflux disease, thrombocytopenia, or bleeding disorders; and no known liver disease, kidney disease, cardiovascular disease, diabetes, or hypertension. Volunteers who met eligibility criteria and were willing to continue in the study (n = 95) were randomized to three treatment arms before starting the supervised 9-month exercise training program: (1) ibuprofen before exercise, placebo after exercise (IBUP/PLAC, *n* = 31), (2) placebo before exercise, placebo after exercise (PLAC/PLAC, *n* = 31), and (3) placebo before exercise, ibuprofen after exercise (PLAC/IBUP, *n* = 33).

### Drug intervention

Participants took two study capsules, one before exercise and one after, on each day of supervised exercise. The capsules were prepared by a local pharmacy (Belmar Pharmacy, Lakewood, CO, USA) and contained either ibuprofen 400 mg or inactive ingredients. All capsules were identical in appearance, with the exception that capsules taken 1 to 2 hours before the exercise sessions were green and those taken immediately after exercise were red. Participants recorded the time that the preexercise capsule was taken when they arrived at the exercise facility. The postexercise pill was taken immediately after completing the exercise session, and the time was recorded. Randomization to the three drug-intervention arms was stratified for use of hormonal contraceptive therapy. The University of Colorado Denver Research Pharmacy managed the randomization process, maintained all drug intervention records, and prepared and dispensed study drug packets.

### Exercise training intervention

All women in the study participated in a 9-month weight-bearing exercise training program. The general structure of the exercise training program was similar to programs used in previous studies that resulted in increases in BMD.(16,17) Participants were instructed to attend at least three supervised exercise sessions per week. The exercise program included weight-bearing endurance exercises (e.g., walking, jogging, rope jumping, and stair climbing/descending), weight-supported endurance exercise that generates relatively high muscle forces (e.g., rowing), box jumps (e.g., step up onto a platform and jump off, 2-ft landing), and upper and lower body resistance exercises. The lower body, biceps, and triceps exercises were performed unilaterally. Exercise was prescribed initially at a moderate intensity (60% to 70% of maximal heart rate, three sets of 12 to 15 repetitions at 60% to 70% of 1-repetition maximum) for a total of 30 min/day. The duration and intensity of exercise were increased gradually on an individualized basis, with exercise prescriptions updated weekly. Box-jump height started at 8 inches and was increased by 2 inches as tolerated to a maximum of 16 inches. The goal was to perform a combination of high-intensity endurance (80% to 85% of maximal heart rate), resistance (three sets of 8 to 10 repetitions at 70% to 80% of 1-repetition maximum), and jumping exercises for a total exercise time of approximately 45 min/day over the last 3 months of the program, not including time to warm up and cool down.

### BMD and body composition

BMD of the total body, lumbar spine (L_2_–L_4_), and proximal femur (total, neck, trochanter, and shaft) was measured by dual-energy x-ray absorptiometry (DXA) on a Hologic Delphi-W instrument (Hologic, Inc., Bedford, MA, USA) at baseline and after 9 months of exercise training. The total-body scan also provided measures of fat mass and fat-free mass. In our laboratory, the coefficients of variation (CVs) for lumbar spine, total-hip, femoral neck, trochanter, and shaft (i.e., subtrochanteric region) BMD are (mean ± SD) 1.2% ± 0.8%, 0.8% ± 0.6%, 1.9% ± 0.9%, 1.5% ± 1.0%, and 1.1% ± 0.6%, respectively. CVs for fat-free mass and fat mass are 1.2% ± 0.8% and 1.8% ± 0.9%, respectively. Calibration procedures included spine phantom scans daily, whole-body phantom scans three times per week, air scans once a week, and tissue bar scans once a month.

### Statistical analyses

Because there have been no previous intervention studies of the effects of regular NSAID use on BMD adaptations to exercise, this was a physiologic study of the effects that occur when there is compliance with the exercise program. Therefore, the primary data analysis was per protocol rather than intent to treat. Compliance was defined as attending at least 80% of the prescribed exercise sessions over the 9-month intervention (i.e., at least 2.4 per week).

Homogeneity across groups at baseline was verified by one-way analyses of variance for age, BMI, weight, fat mass, fat-free mass, and BMD. A chi-square test for equal proportions across groups was applied to ethnicity and hormonal contraception use. The baseline data also were examined for differences by hormonal contraception use with two-group *t* tests, chi-square tests, or exact chi-square tests as appropriate.

The effects of ibuprofen on the BMD responses to exercise training were evaluated by analysis of covariance (ANCOVA), regressing the 9-month change in BMD on baseline BMD and group (IBUP/PLAC, PLAC/IBUP, and PLAC/PLAC). The same method was used to assess the effects of age, change in body weight, and use of hormonal contraception. Although *p* values were not adjusted for multiple comparisons, we protected the type I error rate by requiring a priori that effects be significant for more than one BMD region and by testing for any differences across groups before testing pair-wise differences. Pair-wise comparisons were considered significant only when both the global *F* and the pair-wise comparison were significant at an alpha level .05 or less. Data are presented as mean ± SD, unless otherwise specified.

## Results

Of the 95 volunteers randomized to the drug treatment arms, 73 completed the 9-month exercise program, and 54 completed at least 80% of the prescribed sessions. The attrition and noncompliance rates were higher than expected, but it is unlikely that this was related to the intervention *per se*. Rather, because the study was carried out during a time interval when the university was moving to a new campus, these factors were negatively influenced by relocation of the exercise training facility twice during the study (i.e., from the old campus to an interim off-campus facility and then to the new campus).

The treatment groups in the subset of participants who were compliant with the intervention were well matched on baseline characteristics (Table 1). There were no significant differences in baseline characteristics between users and nonusers of hormonal contraception (Table 2).

**Table 1:** Baseline Characteristics of Compliant Participants

	*N* (%) or mean ± SD and *p* values for comparisons by group				
	
Variable	Category	IBUP/PLAC	PLAC/PLAC	PLAC/IBUP	*p* Value
Hormonal contraceptives	No	9 (52.9)	14 (60.9)	7 (50.0)	0.784
	Yes	8 (47.1)	9 (39.1)	7 (50.0)	
Ethnicity	White	14 (82.4)	20 (87.0)	14 (100.0)	0.404
	Hispanic	2 (11.8)	2 (8.7)	0 (0.0)	
	Other	1 (5.9)	1 (4.3)	0 (0.0)	
Age, years		30.8 ± 6.2	33.2 ± 5.3	32.1 ± 5.0	0.418
BMI, kg/m^2^		24.0 ± 4.5	23.6 ± 2.8	23.6 ± 3.0	0.935
Height, cm		165.0 ± 8.5	167.2 ± 7.8	165.2 ± 5.7	0.580
Weight, kg		66.5 ± 9.3	67.0 ± 9.8	64.9 ± 9.4	0.795
Fat mass, kg		21.5 ± 5.2	22.5 ± 6.7	21.0 ± 7.3	0.775
Fat-free mass, kg		45.0 ± 5.1	44.5 ± 5.1	43.8 ± 5.2	0.821
BMD, g/cm^2^					
Total hip		1.005 ± 0.112	0.982 ± 0.128	0.978 ± 0.116	0.782
Femoral neck		0.896 ± 0.127	0.886 ± 0.114	0.915 ± 0.125	0.793
Trochanter		0.755 ± 0.105	0.760 ± 0.098	0.754 ± 0.127	0.980
Shaft		1.184 ± 0.118	1.150 ± 0.166	1.128 ± 0.130	0.552
Lumbar spine		1.092 ± 0.124	1.130 ± 0.122	1.061 ± 0.152	0.293

IBUP = ibuprofen; PLAC = placebo; BMI = body mass index; BMD = bone mineral density.

**Table 2:** Baseline Characteristics by Use of Hormonal Contraceptive Therapy

	Mean ± SD or *n* (%) and *p* values for comparisons by OC uUse			
	
Variable	Category	Yes	No	*p* Value
Ethnicity	White	20 (83.3)	28 (93.3)	0.653
	Hispanic	3 (12.5)	1 (3.3)	
	Other	1 (4.2)	1 (3.3)	
Age, years		31.0 ± 4.6	33.1 ± 6.0	0.173
BMI, kg/m^2^		22.9 ± 2.2	24.3 ± 4.1	0.119
Height, cm		166.7 ± 6.8	165.4 ± 8.1	0.515
Weight, kg		64.5 ± 8.2	67.8 ± 10.2	0.204
Fat mass, kg		20.2 ± 5.2	23.1 ± 6.9	0.095
Fat-free mass, kg		44.3 ± 4.7	44.6 ± 5.4	0.786
BMD, g/cm^2^				
Total hip		0.986 ± 0.128	0.990 ± 0.113	0.890
Femoral neck		0.901 ± 0.131	0.894 ± 0.112	0.832
Trochanter		0.751 ± 0.112	0.762 ± 0.103	0.714
Shaft		1.154 ± 0.143	1.156 ± 0.144	0.947
Lumbar spine		1.098 ± 0.140	1.102 ± 0.126	0.925

### Exercise intervention

There were no significant differences among the groups in the amount of exercise performed during the intervention. Among those who were compliant, the average exercise frequency was 3.4 ± 0.6, 3.4 ± 0.6, and 3.6 ± 0.6 days per week in the IBUP/PLAC, PLAC/PLAC, and PLAC/IBUP groups, respectively. The average volume of exercise performed by all participants in the second month of the study (after participants had become accustomed to the equipment) and in the eighth month of the study (before final follow-up testing began) is summarized in Table 3. Treadmill walking/running was the most commonly performed endurance exercise.

**Table 3:** Average (Mean ± SD) Volume of Exercise Performed in Months 2 and 8 of the 9-Month Intervention

	Weeks 5–8		Weeks 29–32	
		
	Repetitions	Weight, lb	Repetitions	Weight, lb
Resistance exercises				
Leg extension^a^	39 ± 10	44 ± 16	26 ± 12	52 ± 26
Leg flexion^a^	41 ± 9	44 ± 14	26 ± 13	52 ± 26
Leg press^a^	35 ± 19	86 ± 52	28 ± 15	128 ± 61
Lat pulldown	42 ± 9	107 ± 33	27 ± 14	131 ± 62
Bench press	41 ± 8	63 ± 20	26 ± 13	79 ± 36
Overhead press	40 ± 8	40 ± 13	26 ± 12	49 ± 21
Biceps curl^a^	40 ± 8	22 ± 6	25 ± 13	24 ± 11
Triceps extension^a^	41 ± 8	15 ± 5	27 ± 13	22 ± 12
Seated row	32 ± 20	57 ± 38	26 ± 14	88 ± 42
Box jumps, number/week	110 ± 33		121 ± 60	
Height, in	10.0 ± 0.4		15.7 ± 1.0	
Stairs, flights/week	4.3 ± 3.4		8.6 ± 4.0	
Weight-bearing endurance exercises				
Duration, minutes/week	97.3 ± 40.0		103.0 ± 73.0	
Heart rate, beats/min	154.6 ± 12.2		157.0 ± 11.3	

a Exercises performed unilaterally.

Across the entire 9-month exercise program, the time interval between the preexercise and postexercise drug doses averaged 2.37 ± 0.44, 2.45 ± 0.46, and 2.55 ± 0.46 hours (*p* = .55) for the IBUP/PLAC, PLAC/PLAC, and PLAC/IBUP groups, respectively. Because the postexercise capsule was taken immediately on completion of an exercise session, this indicates that the preexercise capsule typically was taken about 1.5 hours before an exercise session. Pill compliance, assessed as the percentage of prescribed doses based on exercise frequency, was 83% ± 20%, 80% ± 24%, and 86% ± 16% in the IBUP/PLAC, PLAC/PLAC, and PLAC/IBUP groups, respectively.

### Changes in body composition

**Fig. 1:**
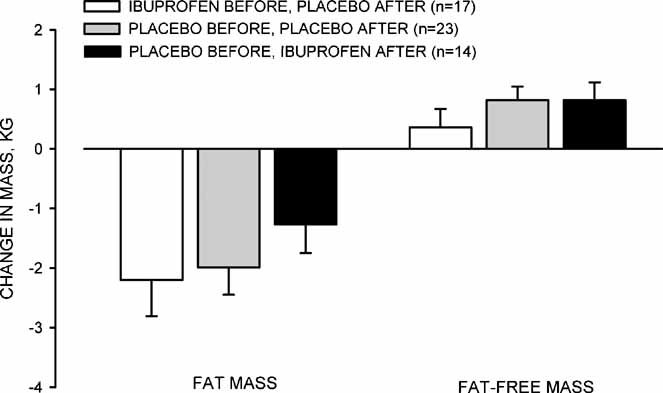
Changes in fat mass and fat-free mass in response to exercise training. Values are mean ± SE.

There were no significant differences among the groups in the changes in fat mass or fat-free mass in response to exercise training (Fig. 1). The changes in fat mass were –2.2 ± 2.5 kg, −2.0 ± 2.2 kg, and −1.3 ± 1.8 kg in the IBUP/PLAC, PLAC/PLAC, and PLAC/IBUP groups, respectively. The respective changes in fat-free mass were 0.4 ± 1.3 kg, 0.8 ± 1.1 kg, and 0.8 ± 1.1 kg.

### Changes in BMD

**Fig. 2:**
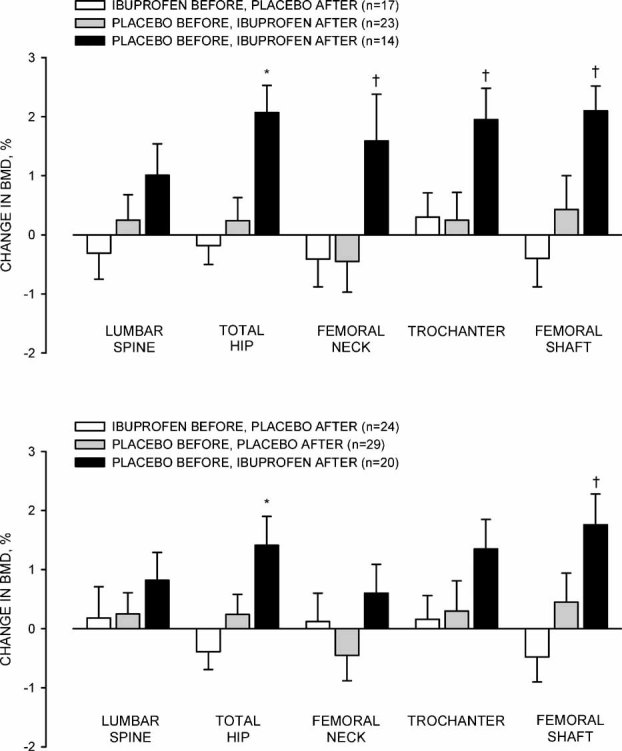
Relative changes (%) in bone mineral density (BMD) adjusted for baseline BMD in response to exercise training in participants who were compliant with the intervention (*top panel*) and in all participants who finished the intervention (*bottom panel*). Values are mean ± SE. ^*^Different from the other groups, *p* ≤ .01. ^†^Different from the other groups, *p* ≤ .05.

As hypothesized, among women who were compliant with the exercise intervention, taking ibuprofen before exercise (IBUP/PLAC) impaired the increases in BMD when compared with taking ibuprofen after exercise (PLAC/IBUP), although the effect at the lumbar spine was not statistically significant (Fig. 2, *top panel*). The differences (mean ± SE, adjusted for baseline BMD) between these two groups in the changes in BMD were 0.013 ± 0.008 g/cm^2^ for the lumbar spine (*p* = .100), 0.020 ± 0.005 g/cm^2^ for the total hip (*p* = .001), 0.018 ± 0.008 g/cm^2^ for the femoral neck (*p* = .020), 0.012 ± 0.006 g/cm^2^ for the trochanter (*p* = .030), and 0.024 ± 0.009 g/cm^2^ for the femoral shaft (*p* = .007). The inclusion of age, change in body weight, or use of hormonal contraception in the analysis model did not change the results. As would be expected, the magnitude of differences between the groups was diminished by the inclusion of participants who were not compliant with exercise (Fig. 2, *bottom panel*). However, the pattern of group differences remained.

In contrast to the hypothesis, the group that received only placebo treatment (PLAC/PLAC) did not have the most robust increases in BMD. Increases in BMD in this group tended to be only slightly, but not significantly, larger than in the IBUP/PLAC group in some regions (Fig. 2, *top panel*). Unexpectedly, the increases in BMD adjusted for baseline BMD were significantly greater in the PLAC/IBUP group than in the PLAC/PLAC group at the femoral neck (difference 0.019 ± 0.007 g/cm^2^, *p* = .010), hip (0.018 ± 0.005 g/cm^2^, *p* = .001), trochanter (0.013 ± 0.005 g/cm^2^, *p* = .020), and femoral shaft (0.019 ± 0.008 g/cm^2^, *p* = .020); the difference at the lumbar spine was not statistically significant (0.006 ± 0.007 g/cm^2^, *p* = .431).

## Discussion

The aim of this study was to determine whether the regular use of ibuprofen before or after exercise sessions influences the BMD adaptations to exercise training. The scientific basis for the study was the evidence from animal studies that the acute increase in bone formation in response to mechanical loading is markedly inhibited when NSAIDs are administered before, but not after, mechanical loading.(7,9,20) Consistent with this evidence, we found that taking ibuprofen before exercise resulted in the least favorable adaptations in BMD. However, in contrast to our expectation that the PLAC/PLAC and PLAC/IBUP groups would have similar increases in BMD, we found that the largest increases occurred in the group that took ibuprofen immediately after completing the exercise sessions.

Ibuprofen and other nonselective and selective COX inhibitors are thought to influence bone metabolism by blocking the production of PGE_2_.(28) The actions of PGE_2_ in bone are complex. The seemingly paradoxical findings that PGE_2_ can both stimulate and inhibit bone formation and resorption may relate to the distribution of the four prostaglandin receptor subtypes in bone.(21) Because the major effects of PGE_2_ in bone are to stimulate both formation and resorption, blocking PGE_2_ production with NSAIDs would be expected to have antiformation and antiresorptive effects.(28) Accordingly, the net effect on BMD could be deleterious or favorable depending both on relative balance between the antiformation and antiresorptive activities and on the state of bone turnover.

Because of the potential antiresorptive actions of COX inhibitors, several animal studies have evaluated whether NSAIDs prevent the bone loss that occurs following ovariectomy as a result of the increase in bone turnover. Indeed, in one of the early studies, 6 weeks of naproxen treatment reduced ovariectomy-related bone loss by approximately 70% in adult rats.(18) However, in a follow-up study, the same investigators found that 12 weeks of naproxen was ineffective in preventing ovariectomy-related bone loss in adult rats.(14) Others have confirmed that NSAIDs are ineffective(13) or only partially effective(10,12) in attenuating ovariectomy-related decreases in bone volume or BMD.

Chronic NSAID use also has been found to cause bone loss in laboratory animals. In ovariectomized adult rats, 24 weeks of indomethacin treatment caused greater decreases in lumbar spine BMD, trabecular bone volume, and trabecular thickness than vehicle treatment.(30) Similarly, treatment of young rats with ibuprofen for 28 days had an independent adverse effect on trabecular bone volume in intact, ovariectomized, ovariectomized estrogen-treated, and ovariectomized tamoxifen-treated animals.(32) Male gonad-intact rats treated with a COX-2 selective inhibitor for 30 days had an increase in bone resorption and a decrease in formation but, paradoxically, BMD was preserved.(31) However, as with the studies of naproxen discussed earlier,(14,18) a longer duration of treatment with a COX-2 selective inhibitor may be necessary to determine the true effects of COX-2 inhibitors on BMD. These studies of laboratory animals highlight the complex and variable effects of NSAIDs on bone metabolism, which may be influenced by the age of the animal, bone turnover state, type and dose of NSAID, and duration of treatment.

In contrast to the variable effects of chronic NSAID treatment on bone metabolism in animals, a consistent finding is that NSAIDs impair the bone-formation response to acute mechanical loading. PGE_2_ was identified as playing a role in mechanical transduction in bone more than 20 years ago.(33) Key evidence that prostaglandins are essential for bone formation came from in vivo mechanical loading experiments in which the expected osteogenic response to mechanical loading was absent when indomethacin was administered.(27) Subsequent studies revealed that the bone-formation response is mediated by COX-2.(2,9) However, in mice lacking COX-2 (i.e., null mutation), a normal bone-formation response to mechanical stimulation was achieved through a compensatory increase in COX-1.(1) This suggests that nonselective COX inhibitors may suppress bone formation to a greater extent than COX-2 selective inhibitors when used chronically.

The timing of NSAID administration relative to mechanical loading has been found to be an important determinant of the bone-formation response. Chow and colleagues were the first to observe that the increase in bone formation in adult rats was suppressed by indomethacin when it was administered before, but not after, mechanical stimulation.(7) This was confirmed subsequently in a study in which a COX-2 inhibitor, NS-398, was administered to rats before or after in vivo mechanical loading of the tibia.(20) NS-398 administered 3 hours or 30 minutes before mechanical loading significantly dampened the bone-formation response, whereas administration 30 minutes after loading did not.

Although there is consistent evidence from studies of animals that the administration of NSAIDs prior to mechanical loading blocks the bone-formation response, a limitation is that the studies have assessed the response to only a single bout of loading. To our knowledge, this study is the first, in humans or animals, to evaluate whether NSAID use, and specifically the timing of use (i.e., before versus after bone-loading exercise), is an important determinant of the adaptation of BMD to repeated mechanical stimulation (i.e., exercise training). Based on the studies of acute loading in animals,(7,20) we hypothesized that taking ibuprofen before exercise sessions would blunt the adaptation in BMD but that taking ibuprofen after exercise sessions would not. The observation that the least favorable changes in BMD tended to occur in the group that took ibuprofen before each exercise training session was interpreted as being consistent with the evidence that COX inhibition prior to loading blocks bone formation.(7,9,20,27) However, the robust increases in BMD in the group that took ibuprofen after exercise were not anticipated. Rather, it was expected that the increases in BMD would be similar in the PLAC/PLAC and PLAC/IBUP groups.

Potential reasons for the smaller than expected BMD changes in the placebo-only group should be considered. One possibility is that because they did not receive any NSAID treatment, the physical discomforts that sometimes occur with vigorous exercise training may have caused them to reduce their physical activity level outside the exercise program. We think that this was unlikely because all participants were instructed to take acetaminophen if they had a need for analgesic therapy. This recommendation was based on the long-standing belief that, in contrast to NSAIDs, the mechanism by which acetaminophen relieves pain does not involve COX suppression. However, recent evidence suggests that acetaminophen does, indeed, act through COX-related mechanisms.(11,15) To our knowledge, the effect of acetaminophen on the bone-formation response to loading has not been studied. However, if effects are similar to those of NSAIDs, and if the group on placebo-only therapy used more acetaminophen than the groups on ibuprofen, then this may have contributed to the small changes in BMD in the placebo group.

Potential reasons for the larger than expected increases in BMD in the group that took ibuprofen after exercise sessions also should be considered. High-intensity endurance and resistance exercise stimulates increases in serum inflammatory cytokines.(22,25,26) For example, serum interleukin 6, which is a potent stimulator of bone resorption,(24) was increased approximately sevenfold 1 hour after a bout of vigorous resistance exercise.(25) In general, intense exercise generates increases in inflammatory cytokines that peak within a few hours of cessation of exercise.(22) If the exercise performed in the current study generated increases in proresorptive cytokines, it is possible that the benefit on BMD of taking ibuprofen immediately after exercise sessions was via the suppression of inflammatory-mediated bone resorption. Further research will be needed to explore such mechanisms.

The bulk of evidence from studies of animals suggests that NSAIDs do not have favorable effects on bone. Accordingly, it might be predicted that chronic NSAID use would be associated with low BMD values in humans. Associations of NSAID use with BMD, bone turnover markers, and/or fracture risk have been evaluated in the Study of Osteoporotic Fractures,(3) Rancho Bernardo Study,(23) Health ABC Study,(4) and Canadian Multicentre Osteoporosis Study.(29) In all these studies, analysis of covariance was used to adjust for an array of parameters likely to be associated with the outcomes of interest. After such adjustments, there was no evidence that NSAID use influenced bone turnover or nonvertebral fracture risk. However, in all four of the cohort studies, there was some evidence for *increased* BMD levels in some groups of NSAID users when compared with nonusers. As an example, in the Rancho Bernardo Study of women aged 44 to 98 years,(23) regular use of proprionic acid NSAIDs was associated with increased BMD levels of the hip (2% to 3%) and lumbar spine (∼9%).(23) This type of favorable association of NSAID use with BMD in humans is seemingly in contrast with the unfavorable effects of NSAIDs on bone metabolism in laboratory animals. However, in the Canadian Multicentre Osteoporosis cohort study,(29) favorable effects of COX-2 selective inhibitors on BMD were apparent only in postmenopausal women and primarily in those on estrogen-based hormone therapy. In contrast, men who used COX-2 selective inhibitors had significantly lower BMD levels than men who did not. Such findings support the concept that bone turnover state may be an important determinant of the effects of NSAIDs on bone metabolism. In high-turnover conditions (e.g., postmenopausal women not on hormone therapy), the antiresorptive effects of NSAIDs may predominate and have a beneficial effect on BMD, whereas in low-turnover conditions (e.g., men), the antiformation effects may predominate and have an adverse effect on BMD.

This study had some limitations. Participants were instructed to use acetaminophen for pain relief when needed, but there is emerging evidence that acetaminophen has COX inhibitory effects.(11,15) No data were collected regarding the use of this medication. Additionally, we did not control for changes in physical activity outside the exercise intervention; between-group differences in nonintervention exercise or activity could have confounded the findings of the study. This was a small study of premenopausal women, and the results may not be applicable to other patient groups with different rates of bone turnover. Because this was the first intervention study of the effects of the timing of NSAID use on adaptations of bone to mechanical loading, many questions remain regarding the potential influence of the type of NSAID, dose, and specific timing of use relative to exercise.

In summary, taking 400 mg of ibuprofen immediately after exercise sessions augmented the beneficial adaptations of BMD to 9 months of exercise training when compared with taking ibuprofen before exercise or with placebo therapy. Because of the common use of over-the-counter NSAIDS among people who exercise regularly, such findings may have important public health ramifications. However, further research is needed both to confirm the results of this first preliminary study of the timing of NSAID use on skeletal adaptations to exercise training and to determine the extent to which the findings can be generalized to other types and doses of NSAIDs and populations other than premenopausal women. It also will be necessary to determine the mechanisms by which timing of NSAID use relative to exercise influences skeletal adaptations.
